# The Role of Non-Steroidal Anti-Inflammatory Drugs in Renal Colic

**DOI:** 10.3390/ph3051304

**Published:** 2010-04-28

**Authors:** Kim Davenport, Elizabeth Waine

**Affiliations:** Bristol Urological Institute, Southmead Hospital, Westbury-on-Trym, Bristol, BS10 5NB, UK; E-Mail: lizziw@hotmail.com (E.W.)

**Keywords:** renal colic, ureteric stone, Non-steroidal anti-inflammatory drugs

## Abstract

NSAIDs provide optimal analgesia in renal colic due to the reduction in glomerular filtration and renal pelvic pressure, ureteric peristalsis and ureteric oedema. Prevention of glomerular afferent arteriolar vasodilatation renders these patients at risk of renal impairment. NSAIDs have the additional benefit of reducing the number of new colic episodes and preventing subsequent readmission to hospital. Despite recent work promoting the use of pharmacological agents to improve stone passage rates, NSAIDs do not appear to reduce the time to stone passage or increase the likelihood of stone passage in renal colic.

## 1. Introduction

Renal colic is a symptom complex characteristic for the presence of a partially or completely obstructing ureteric stone. Typically, the pain is of acute onset, localised to the flank area and may radiate to the groin or genitals. However the pain can be perceived in any organ sharing the same innervation as the kidney and ureter at T11-L1. Other associated symptoms include nausea, vomiting and haematuria. 

The likelihood of spontaneous stone passage is related to the site and size of the stone [1], however stone passage within six weeks is seen in approximately 50% of all ureteric stones [2] and 98% of stones less than 5 mm diameter. Smaller, more distal stones on the right are most likely to pass [3]. 

The pain associated with renal colic results from a combination of responses to the presence of a stone within the ureter. There is an initial stimulation of ureteric peristalsis in an attempt to move the stone. If the stone becomes lodged, the surrounding smooth muscle goes into spasm, which may have a negative effect on the probability of stone passage. Oedema and inflammation further narrow the ureter at the level of the stone. Finally, ureteric peristalsis proximal to the stone increases in an attempt to move the stone [4].

## 2. NSAIDs and Their Mechanism of Action

**Figure 1:**
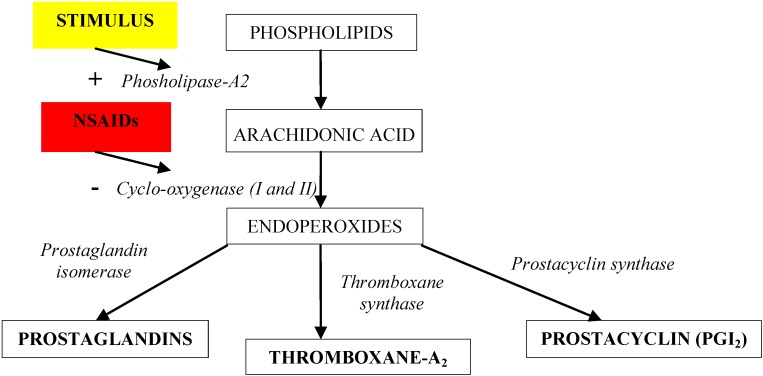
NSAIDs and their mechanism of action

NSAIDs have many unique properties which make them ideal analgesics in renal colic. The predominant analgesic action of NSAIDs in renal colic is through inhibition of prostaglandin synthesis (see Figure 1). Prostaglandin production is stimulated by local irritation by the stone. Prostaglandins promote glomerular afferent arteriolar vasodilatation and increase vascular permeability resulting in increased urine output and renal pelvic pressure. The use of NSAIDs reduces glomerular filtration by up to 35% [5] which in turn reduces renal pelvic pressure and stimulation of stretch receptors. Inhibition of prostaglandin production also reduces ureteric oedema and inflammation enabling better drainage, all of which act to reduce ureteric activity or peristalsis. Furthermore NSAIDs may have a direct effect on ureteric smooth muscle resulting in relaxation [6,7,8].

NSAIDs have their predominant action via inhibition of the cyclo-oxygenase (COX) enzyme which regulates synthesis of prostaglandins and autacoids such as thromboxanes. There are two COX isoforms [9]: COX I is an enzyme found in the stomach and renal blood vessels; COX II is an inflammation related isoform induced by cytokines and inflammation metabolites at the site of the inflammatory stimulus. COX II is present in most cells, including the gastric mucosa, but at low levels. Typically, COX II is upregulated locally in response to an inflammatory stimulus. There are many NSAIDs available; the main differences between them are the incidence and type of side-effects, predominantly gastric irritation and ulceration, renal injury and cardiovascular effects, as a result of differing degrees of cyclo-oxygenase inhibition. Selective inhibition of COX II improves gastrointestinal tolerance but still has a detrimental effect on renal and cardiac function in those with pre-existing disease.

The gastrointestinal side effects are the result of a dual insult on the gastrointestinal tract: the acidic molecules directly irritate the gastric mucosa, and inhibition of COX I reduces the levels of protective prostaglandin, thus increasing gastric acid secretion and reducing bicarbonate and mucous secretion. These predispose patients to gastritis and peptic ulceration. A greater risk of upper gastrointestinal bleed is associated with dual inhibition of the COX isoforms, long half-life and slow release formulations. Overall, the relative risk of an upper gastrointestinal bleed with NSAID is 4.5 (3.8–5.3), however ibuprofen has the lowest relative risk (2.6) whilst the highest relative risk is seen with Ketorolac (14.54). Indomethacin and diclofenac have risks of 5.4 and 3.98 respectively [10]. Comparative studies have failed to demonstrate significant differences in efficacy between diclofenac, indomethacin and ketorolac [11,12]. 

NSAIDs potentially interfere with the renal auto-regulatory response to obstruction by decreasing renal blood flow [5]. This is generally well tolerated in healthy individuals, but renal failure may be induced in those patients with pre-existing renal disease, dehydration, cirrhosis, or with the concurrent use of contrast agents and nephrotoxic drugs. Prostaglandins cause vasodilatation of the glomerular afferent arterioles and are essential to maintain normal glomerular perfusion and glomerular filtration rate (GFR). This is particularly important in renal failure where the kidney is trying to maintain renal perfusion pressure by elevated angiotensin II levels. At these elevated levels, angiotensin II constricts the afferent arteriole in addition to constriction of the efferent arteriole. Prostaglandins serve to dilate the afferent arteriole; by blocking this prostaglandin-mediated effect, NSAIDs cause unopposed constriction of the afferent arteriole and decrease renal perfusion pressure. Nausea and vomiting often seen in patients with renal colic may cause dehydration which will further contribute to renal impairment. The risk of acute renal injury increases with decreased selectivity; COX II inhibitors have the lowest risk followed by diclofenac, ibuprofen, naproxen, indomethacin and ketorolac respectively [13].

In patients with pre-existing cardiac disease, the use of NSAIDs for renal colic, may increase the risk of promoting heart failure and cardiac decompensation by increasing peripheral systemic resistance and reducing renal perfusion in patients with impaired ventricular function and compensatory increased vasodilator prostaglandins. Reduced renal blood flow, glomerular filtration and sodium excretion results in fluid overload and further increases systemic resistance increasing the risk of heart failure in susceptible patients [14,15].

The risk of serious vascular events is also increased. Selective COX II inhibitors are associated with a 1.4-fold increased risk of serious vascular events, largely due to a twofold increased risk of myocardial infarction. High dose ibuprofen (800 mg three times daily) and high dose diclofenac (75 mg twice daily) are each associated with an increased risk of vascular events, but the risk with naproxen (500 mg twice daily) is substantially smaller. This is thought to be due to concurrent inhibition of platelet aggregation [16]. 

## 3. The Role of NSAIDs in Providing Analgesia in Renal Colic

NSAIDs are known to be as effective as opioids at relieving the pain of acute renal colic. The only disadvantage is the delayed onset of action following oral or rectal NSAIDs as opposed to intravenous morphine; however by 20–30 min there is no significant difference in pain scores [17]. Intravenous preparations are available with a faster onset of action but this is accompanied by a higher risk of side-effects (Table 1) [18], including nausea, vomiting, a sensation of heat or tension across the chest, giddiness, tiredness and general malaise. 

**Table 1:** The mean pain score (as a percentage of that before treatment) after 50 mg intravenous or 100 mg rectal indomethacin [18].

		Mean Pain Score %	
		Intravenous Indomethacin	Rectal Indomethacin
Time from Administration (min)	10	54	73
	20	29	50
	30	16	29
Side effects %		55	37

In a meta-analysis by Holdgate [19], those receiving NSAIDs were less likely to need rescue analgesia, less likely to experience vomiting (5.8% *vs.* 19.5%) and achieved greater reductions in pain scores.

As well as relieving acute pain, oral diclofenac and oral/rectal indomethacin have both been shown to be effective at reducing the number of new colic episodes. This has been shown to significantly reduce further admissions to hospital by 28–57% (Table 2) [20,21,22].

**Table 2:** The use of NSAIDs for the prophylaxis of pain and promotion of stone passage in acute renal colic.

	Laerum 1995 [20]		Grenabo 1984 [21]		Kapoor 1985 [22]	
	Diclofenac 50 mg PO tds	Placebo tds	Indomethacin 25mg PO bd + 100 mg PR nocte	Placebo bd + nocte	Indomethacin 50 mg PR tds	Placebo tds
n	41	39	37	41	13	13
Mean stone size	78% <6 mm	97% <6 mm	2.9 mm	2.8 mm	3.4 mm	3.1 mm
Readmission Rate	10%	67%	11%	39%	0%	38%
Mean stone passage rate	68% at 3 weeks	74% at 3 weeks	59% at 1 week	61% at 1 week	11/13	10/13
Mean interval to passage	3 days	3.8 days	N/A	N/A	3.4 days	3.7 days

Cole [23] demonstrated a beneficial role in the prevention of renal colic following extracorporeal shock wave lithotripsy (SWL). In this placebo controlled randomised trial, Indomethacin suppositories significantly reduced post treatment pethidine requirements (6 of 28 patients required 10 doses as compared with 18 of 33 patients requiring 41 doses, p < 0.01). NSAID are frequently used as analgesia during SWL. Aspirin may increase the risk of bleeding and peri-renal haematomas during treatment, especially in the presence of uncontrolled hypertension [24], however there is no evidence that other NSAID increase this risk.

## 4. The Role of NSAIDs in the Promotion of Stone Passage

There is much debate as to whether peristalsis is necessary to promote stone passage or whether relaxation of ureteric smooth muscle has a greater effect upon stone movement. It has been stipulated in the past that ureteric peristalsis is essential to allow spontaneous stone passage. However, irritation and stretch stimulation of the ureter by the stone may result in increased uncoordinated peristalsis [25,26], and thus may actually hinder stone passage. In a canine *in vivo* model of acute obstruction, the mean peristaltic rate, baseline pressure and peak pressures above the level of obstruction were all shown to increase significantly [20]. Conversely below the level of obstruction, the mean peristaltic rate remained unchanged but the baseline and peak pressures generated were both significantly reduced. Control of this increased, uncoordinated ureteric activity may help to normalise peristalsis and promote spontaneous stone passage. 

In the laboratory, NSAIDs appear to reduce ureteric activity and in some cases, ablate all ureteric activity. Lennon [7] used canine proximal ureter to record the response diclofenac had on spontaneous ureteric contractions. A reduction in the rate of spontaneous contraction was seen, with abrupt cessation of all spontaneous activity following the application of diclofenac at 10^-5^ M concentration. Sivrikaya [8] reported a mean reduction in proximal ureteric smooth muscle tension of 43 ± 9% following the application of 10^-5^ M diclofenac. Ureteric relaxation reached maximum at 20 minutes. 

Cole [6] used single pulse electrical field stimulation to produce phasic contractions in distal and proximal circular strips of human ureter. The application of 10^-5^ M diclofenac almost completely abolished activity in all but one preparation; however this effect was not immediate, taking 5–20 minutes to occur. 

Ureteric muscle is known to fatigue with prolonged stimulation and therefore it is difficult to determine whether the relaxant effects in these studies were drug related or due to muscle fatigue. *In vivo* assessment of the effect NSAIDs have on ureteric peristalsis is difficult; however diclofenac does not appear to effect ureteric contraction frequency [27].

Despite the *in vitro* reduction of ureteric activity, this has not translated to an increase in spontaneous stone passage rates or a reduction in the time to stone passage in clinical trials (see Table 2). Laerum [20] randomised patients to 7 days diclofenac 50 mg tds (n = 41) or placebo tds (n = 39). At 3 weeks follow up, 68% of those receiving diclofenac had passed their stones as compared with 74% in the placebo group. The mean time to passage was also similar in both groups, 3 days *vs.* 3.8 days. However stone size is unclear and there are no p values with regards time to stone passage or stone passage rates.

These results are consistent with other studies using indomethacin [21,22]. Kapoor [21] recruited and randomised patients to indomethacin suppositories (n = 13) *vs.* placebo (n = 13). The mean time interval to stone passage was slightly lower in the indomethacin group at 82 hours as opposed to 89 hours for placebo (p > 0.1), however the groups were small and the time to stone passage was short which would make it difficult to identify a significant difference. Grenabo [22] used indomethacin (n = 37) *vs.* placebo (n = 41) for 7 days and found the rate of stone passage within these 7 days was not influenced by indomethacin (22/37 and 25/41 cases). 

## 5. Conclusions

NSAIDs provide excellent analgesia in renal colic, but should be used with care in patients at risk of renal impairment, cardiac failure and gastric ulceration. They are highly effective in reducing the number of new colic episodes and readmissions to hospital; however they do not appear to have any effect on the time to stone passage or the likelihood of stone passage in renal colic. 

## References

[1] Coll D.M., Varanelli M.J., Smith R.C. (2002). Relationship of spontaneous stone passage of ureteral calculi to stone size and location as revealed by unenhanced helical CT. Am. J. Roentgenol..

[2] Miller O.F., Kane C.J. (1999). Time to stone passage for observed ureteral calculi: A guide for patient education. J. Urol..

[3] Painter D.J., Keeley F.X. (2001). New concepts in the treatment of ureteral calculi. Curr. Opin. Urol..

[4] Crowley A.R., Byrne J.C., Darracott Vaughan E., Marion D.N. (1990). The effect of acute obstruction on ureteral function. J. Urol..

[5] Perlmutter A., Miller L., Trimble L.A., Marion D.N., Vaughan E.D., Felson D. (1993). Toradel, an NSAID used for renal colic, decreases renal perfusion and ureteral pressure in a canine model of unilateral ureteral obstruction. J. Urol..

[6] Cole R.S., Fry C.H., Shuttleworth K.E.D. (1988). The action of the prostaglandins on isolated human ureteric smooth muscle. Br. J. Urol..

[7] Lennon G.M., Bourke J., Ryan P.C., Fitzpatrick J.M. (1993). Pharmacological options for the treatment of acute ureteric colic. Br. J. Urol..

[8] Sivrikaya A., Celik O.F., Sivrikaya N., Ozgur G.K. (2003). The effect of diclofenac sodium and papaverine on isolated human ureteric smooth muscle. Int. Urol. Neph..

[9] Nakada S.Y., Jerde T.J., Bjorling D.E., Saban R. (2000). Selective cyclooxygenase-2 inhibitors reduce ureteral contraction *in vitro*: A better alternative for renal colic?. J. Urol..

[10] Gonzalez E.L., Patrignani P., Tacconelli S., Rodriquez L.A. (2010). Variability of risk of upper gastrointestinal bleeding among nonsteroidal anti-inflammatory drugs. Arthritis Rheum..

[11] Riedemann P.J., Bersinic S., Cuddy L.J., Torrance G.W., Tugwell P.X. (1993). A study to determine the efficacy and safety of tenoxicam versus piroxicam, diclofenac and indomethacin in patients with osteoarthritis: a meta-analysis. J. Rheumatol..

[12] Cohen E., Hafner R., Rotenberg Z., Fadilla M., Garty M. (1998). Comparison of ketorolac and diclofenac in the treatment of renal colic. Eur. J. Clin. Pharmacol..

[13] Lafrance J.P., Miller D.R. (2009). Selective and non-selective non-steroidal anti-inflammatory drugs and the risk of acute kidney injury. Pharmacoepidemiol. Drug. Saf..

[14] Bleumink G.S., Feenstra J., Sturkenboom M.C., Stricker B.H. (2003). Non-steroidal anti-inflammatory drugs and heart failure. Drugs.

[15] Dzau V.J., Packer M., Lilly L.S., Swartz S.L., Hollenberg N.K., Williams G.H. (1984). Prostaglandins in severe congestive heart failure. N. Engl. J. Med..

[16] Kearney P.M., Baigent C., Godwin J,, Halls H., Emberson J.R., Patrono C. (2006). Do selective cyclo-oxygenase-2 inhibitors and traditional non-steroidal anti-inflammatory drugs increase the risk of atherothrombosis? Meta-analysis of randomised trials. BMJ..

[17] Cordell W.H., Larson T.A., Lingeman J.E., Nelson D.R., Woods J.R., Burns L.B., Klee L.W. (1994). Indomethacin suppositories *versus* intravenous titrated morphine for the treatment of ureteral colic. Ann. Emerg. Med..

[18] Nissen I., Birke H., Olson J.B., Würtz E., Lorentzen K., Salomon H., Lynge P., Fly P., Jørgensen T.H., Svane S. (1990). Treatment of ureteric colic: intravenous *versus* rectal administration of indomethacin. Br. J. Urol..

[19] Holdgate A., Pollock T. (2004). Systematic review of the relative efficacy of non-steroidal anti-inflammatory drugs and opioids in the treatment of acute renal colic. BMJ..

[20] Laerum E., Omundsen O.E., Gronseth J.E., Christianson A., Fagertun H.E. (1995). Oral diclofenac in the prophylactic treatment of recurrent renal colic: A double blind comparison with placebo. Eur. Urol..

[21] Grenabo L., Holmlund D. (1984). Indomethacin as prophylaxis against recurrent ureteral colic. Scand. J. Urol. Nephrol..

[22] Kapoor D., Weitzel S., Mowad J., Melanson S., Gillen J. (1989). Use of indomethacin suppositories in the prophylaxis of recurrent ureteral colic. J. Urol..

[23] Cole R.S., Palfrey E.L.H., Smith S.E., Shuttleworth K.E.D. (1989). Indomethacin as prophylaxis against ureteral colic following extracorporeal shock wave lithotripsy. J. Urol..

[24] Labanaris A.P., Kuhn R., Schott G.E., Zugor V. (2007). Perirenal haematomas induced by extracorporeal shock wave lithotripsy (ESWL). Therapeutic management. Sci. World J..

[25] Rose J.G., Gillenwater J.Y. (1973). Pathophysiology of ureteral obstruction. Am. J. Physiol..

[26] Davenport K., Timoney A.G., Keeley F.X. (2006). A comparative *in vitro* study to determine the beneficial effect of calcium-channel and alpha (1)-adrenoceptor antagonism on human ureteric activity. BJU. Int..

[27] Davenport K., Timoney A.G., Keeley F.X. (2007). Effect of smooth muscle relaxant drugs on proximal human ureteric activity *in vivo*: a pilot study. Urol. Res..

